# Prehospital risk factors of mortality and impaired consciousness after severe traumatic brain injury: an epidemiological study

**DOI:** 10.1186/1757-7241-22-1

**Published:** 2014-01-07

**Authors:** Sophia Tohme, Cecile Delhumeau, Mathias Zuercher, Guy Haller, Bernhard Walder

**Affiliations:** 1 Division of Anaesthesiology, University Hospitals of Geneva, Geneva, Switzerland; 2 Department of Anaesthesia and Intensive Care Medicine, University Hospital Basel, Basel, Switzerland; 3 Division of Clinical Epidemiology, University Hospitals of Geneva, Geneva, Switzerland

**Keywords:** Out-of-hospital emergency medical services, Prehospital emergency medicine, Head injury, Short term outcome

## Abstract

**Background:**

Severe traumatic brain injury (TBI) is a significant health concern and a major burden for society. The period between trauma event and hospital admission in an emergency department (ED) could be a determinant for secondary brain injury and early survival. The aim was to investigate the relationship between prehospital factors associated with secondary brain injury (arterial hypotension, hypoxemia, hypothermia) and the outcomes of mortality and impaired consciousness of survivors at 14 days.

**Methods:**

A multicenter, prospective cohort study was performed in dedicated trauma centres of Switzerland. Adults with severe TBI (Abbreviated Injury Scale score of head region (HAIS) >3) were included. Main outcome measures were death and impaired consciousness (Glasgow Coma Scale (GCS) ≤13) at 14 days. The associations between risk factors and outcome were assessed with univariate and multivariate regression models.

**Results:**

589 patients were included, median age was 55 years (IQR 33, 70). The median GCS in ED was 4 (IQR 3-14), with abnormal pupil reaction in 167 patients (29.2%). Median ISS was 25 (IQR 21, 34). Three hundred seven patients sustained their TBI from falls (52.1%) and 190 from a road traffic accidents (32.3%). Median time from Out-of-hospital Emergency Medical Service (OHEMS) departure on scene to arrival in ED was 50 minutes (IQR 37-72); 451 patients had a direct admission (76.6%). Prehospital hypotension was observed in 24 (4.1%) patients, hypoxemia in 73 (12.6%) patients and hypothermia in 146 (24.8%). Prehospital hypotension and hypothermia (apart of age and trauma severity) was associated with mortality. Prehospital hypoxemia (apart of trauma severity) was associated with impaired consciousness; indirect admission was a protective factor.

**Conclusion:**

Mortality and impaired consciousness at 14 days do not have the same prehospital risk factors; prehospital hypotension and hypothermia is associated with mortality, and prehospital hypoxemia with impaired consciousness.

## Background

Severe traumatic brain injury (TBI) is a worldwide health concern and a major burden for society [1,2] related to long hospital stays, persistence of physical handicap and neuro-psychological alterations, and a long period of working inability if there is any return to work at all [3]. It is one of the main contributors of life-year loss due to its high mortality of 30 to 70% in a younger population [4,5]. It also accounts for about two thirds of all trauma related fatalities [4,6]. Severe TBI has a high mortality rate in the early period and survivors present many major, in-hospital complications [7]. Surviving patients after severe TBI suffer also from a lower life expectancy than the general population [8].

The pathophysiology of severe TBI can be divided into primary and secondary brain injury [9]. Primary injury results from the direct, physical brain trauma with tissue distortion, shearing, vascular injury, and cell destruction probably related to rotational acceleration and deceleration inertial forces [10]. Secondary brain injury is related to destructive inflammation and biochemical changes [11,12]. Secondary injury onsets within minutes of primary injury, may last for several days and contributes to final outcome [13,14]. Primary and secondary brain injuries induce cerebral oedema and bleeding. Factors associated with secondary brain injury are arterial hypotension, hypoxemia and hypothermia; these adverse events are associated with increased mortality and poor outcome [15].

Out-of-hospital emergency medical systems (OHEMS) should ensure the shortest delay possible between the sustainment of the trauma and the patient’s admission to a trauma center and decrease the factors associated with secondary brain injury [16]. Despite the potential benefits of early interventions such as oxygen administration with or without orotracheal intubation and mechanical ventilation, or fluid administration and heat preservation, the evidence for their efficacy is controversial [9]. In clinical practice it is difficult to estimate secondary brain injury related to prolonged prehospital periods, prehospital hypotension, prehospital hypoxemia, or prehospital hypothermia. Death [17] and impaired consciousness of survivors [18] several days after the TBI can be used as clinical surrogates of early secondary brain injury [19-21]. Survival with regained consciousness with execution of commands several days after the TBI can be considered as an early, clinical surrogate of neural network re-functioning [22] and is a predictor of the long term functional outcome [23].

The aim of this study was to investigate the relationship between prehospital factors associated with secondary brain injury and the outcomes of mortality and impaired consciousness at fourteen days.

## Methods

### Design of study

We conducted a prospective epidemiological cohort study with follow-up of patients with severe TBI from the time of accident until 14 days or earlier death. This study is a part of the Swiss National Cohort of severe TBI entitled “Patient-relevant Endpoints after Brain Injury from Traumatic Accidents” (PEBITA; http://www.pebita.ch). Demographic data of this study has been published recently [24].

Switzerland has a surface of 41'285 km^2^ and a population of 7.95 Mio (2011). Half of the population lives in and around larger cities (3.7 Mio). The country’s population density is 193 inhabitants per km^2^. About 130 OHEMS (104 certified OHEMS) are available [3.1 (2.5)/ 1’000 km^2^ or 1.6 (1.3)/100’000 population]. Eighteen helicopter EMS cover the area of the country [0.4/1’000 km^2^ or 0.2/100’000 population]. Twelve governmental defined trauma centers with dedicated emergency physicians manage patients with severe TBI together with neurosurgeons and anesthesiologists based on local clinical pathways.

The study was approved by the ethics committees of the participating trauma centers (Ethics committee of Geneva, protocol NAC 07-013, approval: 7.5.2007). As a result of their neurological condition, patients were unable to give informed consent before enrolment. The local study coordinators contacted their legal representatives (proxies) to inform them of the study within 14 days following the TBI. Both patients and/or proxies received detailed written information on the study and were asked for consent. In the case of withdrawal, further follow-up was discontinued. Complying with a patient’s request, the collected data was removed from the database and destroyed.

### Included patients

We included patients ≥16 years having sustained severe TBI from both blunt and penetrating trauma in Switzerland. Severe TBI was defined by an Abbreviated Injury Scale score of head region (HAIS) of more than 3; AIS is a six point score of trauma severity [25]. HAIS was assessed based on the diagnoses that neurosurgeons or radiologists in charge and established after computer tomography imaging of the head. The worst CT scan in the first 24 hours was assessed using a standardized data sheet based on the HAIS and Marshall classification [26]. Patients who died before neurosurgical or radiological diagnosis were included if the history of trauma and trauma signs of severe head injury were documented by the OHEMS. Patients with unclear brain trauma history (for instance, comatose patients found on a public area without observation by bystanders) or no signs of brain trauma (for instance, fatal multi-trauma patients with abdominal and thoracic injuries without visual injuries to the head) were excluded. We excluded patients without body temperature documentation at hospital admission.

The GCS was not used as an inclusion criterion due to its large inter-rater variability, in particular, in the emergency setting [27]. In a pilot study we observed that of 118 potentially eligible participants 12 could not be included since the inclusion criterion “initial GCS <9 without sedation” was not met [5]. However, these patients had injuries compatible with severe TBI according to the AIS criterion.

### Outcome measures

Mortality at 14 days.

Impaired consciousness (estimated with the GCS) at 14 days; impaired consciousness was defined as GCS ≤13 and consciousness was defined as GCS 14 and 15.

### Potential predictors

i. *Patients’ characteristics:* age

ii. *Initial neuro-physiological variables:* Glasgow Coma Scale (GCS; comatose patients were defined as presenting a GCS <9), pupil reaction

iii. *Trauma mechanism:* road traffic accidents, falls and other mechanisms. Blunt/penetrating trauma was not investigated in details because more than 95% of injuries were blunt trauma. Results on the few penetrating trauma were reported separately.

iv. *Trauma severity:* The Injury Severity Score (ISS) within the 24 hours following the injury event was used which included concomitant injuries. [28]

v. Prehosptial time, e.g. the period of time from OHEMS departure from the scene of injury to its arrival in the emergency department (ED) in trauma centre and direct or indirect admission to trauma centre were collected.

vi. Prehospital arterial hypotension was defined as systolic blood pressure <90 mmHg measured at any time point until hospital admission including the hospital arrival value.

vii. Prehospital hypoxia was defined as a pulse oximeter oxygen saturation <90% at any time point until hospital admission including hospital arrival value.

viii. Prehospital hypothermia was defined as a temperature ≤35.0°C at hospital admission.

### Statistics

Qualitative variables such as sex, pupil reaction, HAIS, mechanism of accident, hypotension, hypoxemia, hypothermic and direct or indirect admission to ED were summarized using percentages for the entire cohort. Quantitative variables such as age, GCS in ED, ISS, and time from OHEMS departure on scene to arrival in ED in trauma centre were described by their median and interquartile ranges (IQRs) (25^th^ to 75^th^ percentile) for the entire cohort.

Descriptive statistics were conducted for the following subgroups: Survivors versus non-survivors at 14 days and impaired consciousness (GCS ≤13) for survivors versus consciousness (GCS 14 and 15) for survivors at 14 days. Differences between two groups were assessed by non-parametric Wilcoxon t-tests with an alpha threshold of 5% for quantitative variables, and by χ^2^ tests with an alpha threshold of 5% for qualitative variables.

In order to evaluate the association between prehospital risk factors and outcome at 14 days we performed a Cox regression (survival) or a logistic regression (impaired consciousness). Age, GCS in ED, pupil reaction, ISS, mechanism of accident, arterial hypotension, hypoxemia, hypothermic, prehospital time and direct or indirect admission to ED were first included in a univariate Cox regression or logistic regression. All variables with p <0.200 in the univariate model were entered in a multivariate model.

All statistical analyses were performed using STATA Release 12 · 0 (Stata Statistical Software: Release 12 · 0, Stata Corporation, College Station, USA).

## Results

### Demographic data

**Figure 1:**
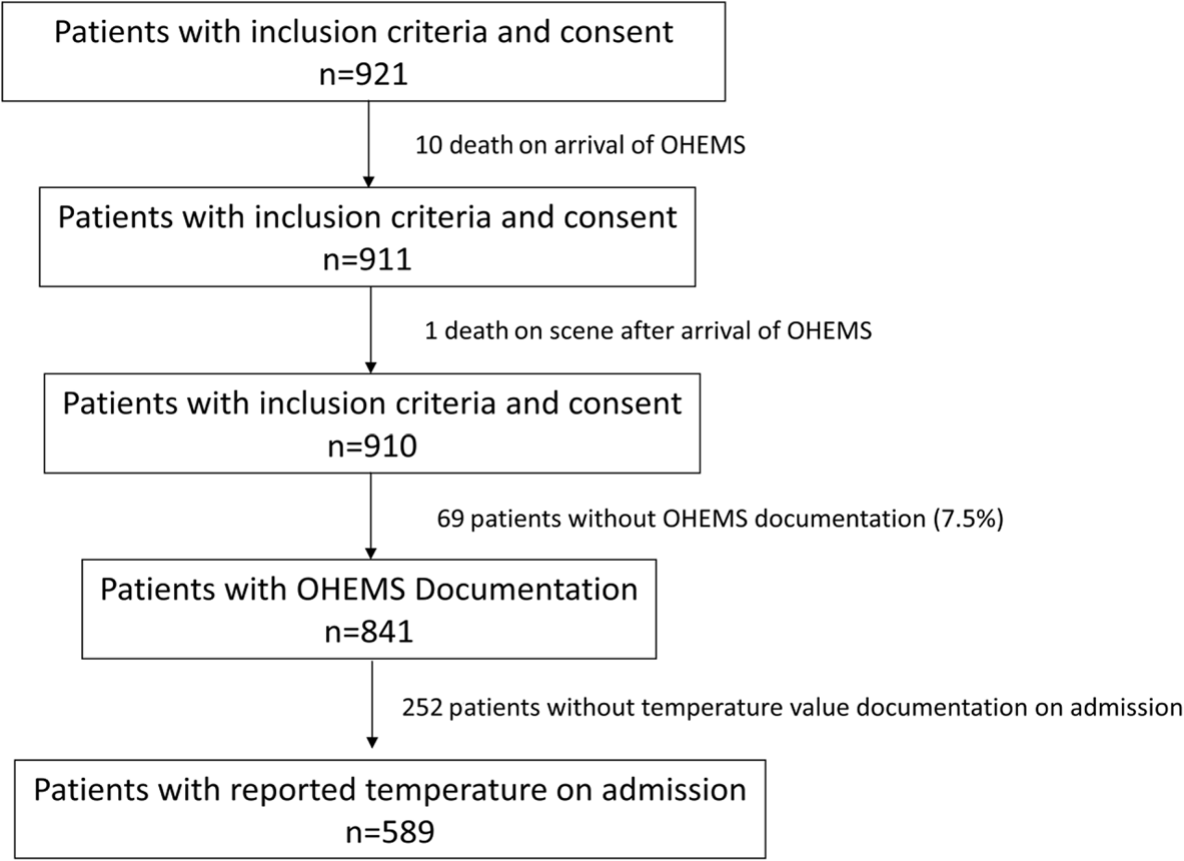
Flow chart of enrolled and included patients with severe TBI.

Five hundred and eighty-nine patients were included (Figure 1). The median age of the patients was 55 years (IQR 33-70). One hundred forty six patients (24.8%) were women. The median GCS on-scene was 9 (IQR 4-14), and 4 (IQR 3-14) in the ED. In the ED, 343 patients (59.0%) had a GCS <9. Abnormal pupil reaction on scene was observed in 128 patients (25.8%), and in 167 patients (29.2%) in the ED. The majority of patients sustained a blunt trauma (562 out of 589; 95.4%). Two hundred forty-seven patients out of 589 had a HAIS of 4 (41.9%) and 330 a HAIS of 5 (56.0%). The median ISS was 25 (IQR 21-34). Three hundred and seven patients sustained their TBI from a fall (52.1%) and 190 from a road traffic accidents (32.3%). Twenty-seven patients with a penetrating trauma (4.6%) were younger (median 35; IQR 26-56) with a lower GCS in the ED (median 3; IQR 3-4) and a higher ISS (median 29; IQR 25-41).

The median time from OHEMS departure on scene to its arrival in the ED was 50 minutes (IQR 37-72). Four hundred fifty-one patients had a direct admission (76.6%); the median time was 46 minutes (IQR 35-58). One hundred and thirty eight patients had an indirect admission (23.4%); the median time was 198 minutes (IQR 143-297). Prehospital arterial hypotension was observed in 24 (4.1%) patients. Prehospital hypoxemia was observed in 73 (12.6%) patients. Prehospital hypothermia was observed in 146 (24.8%) patients.

Five hundred forty-three patients were managed by an OHEMS [of those, 209 with a helicopter OHEMS (38.5%)]; 44 patients were admitted to the trauma centre without OHEMS (7.5%). Two thirds of the patients (317 out of 467 patients, where this data was available) were treated by a certified prehospital physician. Two hundred forty-five patients (45.6%) had a prehospital intubation and 481 had prehospital intravenous fluids (84.4%).

### Outcome at 14 days

During 3 839 person-days of follow-up, there were 159 deaths. Survival rate was 73.0% (430 out of 589 patients) at 14 days (Table 1); survival rate of patients with penetrating trauma was 40.7% (9 out of 27).

**Table 1:** Description of surviving and not surviving patients at 14 days

	**Total**		**Survivors**	**Non survivors**	
	**N missing**	**N (%)**	**N (%)**	**N (%)**	**P values**
		**N = 589**	**N = 430**	**N = 159**	
**Patient-related risks**					
Female	0	146 (24.8)	108 (25.1)	38 (23.9)	0.761
Age (median, IQR)	0	55 (33-70)	52 (31-67)	62 (42-80)	<0.0001
GCS in ED (median, IQR)	8	4 (3-14)	9 (3-14)	3 (3-4)	<0.0001
Abnormal pupil reaction in ED	17	167 (29.2)	75 (18.0)	92 (59.0)	<0.0001
ISS (median, IQR)	0	25 (21-34)	25 (20-32)	25 (25-38)	<0.0001
HAIS 4	0	247 (41.9)	223 (51.9)	24 (15.1)	<0.0001
HAIS 5		330 (56.0)	207 (48.1)	123 (77.4)	
HAIS 6		12 (2.0)	0 (0)	12 (7.6)	
Falls	0	307 (52.1)	218 (50.7)	89 (56.0)	0.495
Road traffic accidents		190 (32.3)	144 (33.5)	46 (28.9)	
Others mechanisms		92 (15.6)	68 (15.8)	24 (15.1)	
**Treatable prehospital risks**					
Hypotension (syst BP <90)	1	24 (4.1)	12 (2.8)	12 (7.6)	0.009
Hypoxemia	9	73 (12.6)	41 (9.6)	32 (20.8)	<0.0001
Hypothermic	0	146 (24.8)	90 (20.9)	56 (35.2)	<0.0001
**Intervention-related risks**					
Time (median, IQR) [min]	137	50 (37-72)	50 (38-75)	48 (36-65)	0.264
Direct admission	0	451 (76.6)	322 (74.9)	129 (81.1)	0.112
Indirect admission		138 (23.4)	108 (25.1)	30 (18.9)	

The median GCS of survivors at 14 days was 15 (IQR 13-15). Impaired consciousness at 14 days was observed in 104 out of 411 survivors (25.3%; missing: 19) (Table 2). An abnormal pupil reaction was observed at 14 days in 20 out of 430 survivors.

**Table 2:** Description of surviving patients with impaired consciousness (GCS <14) and regained consciousness (GCS14 and 15) at 14 days

	**Total**		**GCS 14/15**	**GCS ≤13**	
	**N missing**	**N (%)**	**N (%)**	**N (%)**	**P values**
		**N = 411**	**N = 307**	**N = 104**	
**Patient-related risks**					
Female	0	103 (25.1)	81 (26.4)	22 (21.2)	0.287
Age (median, IQR)	0	53 (34-68)	53 (33-69)	47 (25-64)	0.027
GCS in ED (median, IQR)	6	9 (3-14)	13 (3-15)	3 (3-4)	<0.0001
Abnormal pupil reaction in ED	13	69 (17.3)	38 (12.8)	31 (31.0)	<0.0001
ISS (median, IQR)	0	25 (20-32)	25 (17-29)	29 (25-38)	<0.0001
HAIS 4	0	216 (52.6)	184 (59.9)	32 (30.8)	<0.0001
HAIS 5		195 (47.5)	123 (40.1)	72 (69.2)	
Falls	0	205 (49.9)	163 (53.1)	42 (40.4)	0.009
Road traffic accidents		139 (33.8)	91 (29.6)	48 (46.2)	
Others mechanisms		67 (16.3)	53 (17.3)	14 (13.5)	
**Treatable prehospital risks**					
Hypotension (syst BP <90)	0	12 (2.9)	7 (2.3)	5 (4.8)	0.009
Hypoxemia	4	36 (8.9)	14 (4.6)	22 (21.2)	<0.0001
Hypothermic	0	83 (20.2)	51 (16.6)	32 (30.8)	0.002
**Intervention-related risks**					
Time (median, IQR) [min]	107	50 (38-75)	57 (43-75)	49 (36-77)	0.092
Direct admission	0	306 (74.5)	216 (70.6)	90 (86.5)	0.001
Indirect admission		105 (25.6)	91 (29.4)	14 (13.5)	

### Prehospital risk factors for mortality at 14 days

In the univariate model, 7 risk factors were associated with a higher risk of death at 14 days (age, GCS <9 in ED, abnormal pupil reaction, ISS ≥25, prehospital arterial hypotension, prehospital hypoxemia and prehospital hypothermia; Table 3); not associated were trauma mechanisms, prehospital time, and type of admission.

**Table 3:** Risk factors of death at 14 days

	**Univariate model**		**Multivariate model**	
	**Hazard ratio (95% CI)**	**P values**	**Hazard ratio (95% CI)**	**P values**
**Patient-related risks**				
Age	1.02 (1.01-1.03)	<0.0001	1.03 (1.02-1.04)	0.0001
GCS ≥9 in ED	1		1	
GCS <9 in ED	3.98 (2.57-6.14)	<0.0001	2.08 (1.25-3.46)	0.005
Normal pupil reaction in ED	1		1	
Abnormal pupil reaction in ED	4.00 (2.51-5.51)	<0.0001	2.47 (1.72-3.54)	<0.0001
ISS <25	1		1	
ISS ≥25	4.03 (2.37-6.85)	<0.0001	2.62 (1.48-4.63)	0.0001
Falls	1			
Road traffic accidents	0.80 (-1.24; 0.22)	0.216		
Others mechanisms	0.90 (-0.47; 0.64)	0.636		
**Treatable prehospital risks**				
Hypotension (syst BP <90)	2.71 (1.50-4.89)	0.001	2.13 (1.12-4.04)	0.001
Hypoxemia (SpO2 <90%)	1.93 (1.31-2.85)	0.001	1.44 (0.95-2.17)	0.083
Hypothermia (<35.0°C)	1.80 (1.30-2.49)	<0.0001	1.42 (1.00-2.01)	0.049
**Intervention-related risks**				
Prehospital time	1.00 (0.99-1.00)	0.079		
Direct admission	1		1	
Indirect admission	0.71 (0.48-1.058)	0.092	0.88 (0.58-1.34)	0.552

In the multivariate model, 6 risk factors were independently associated with a higher risk of death at 14 days: age, GCS <9 in ED, abnormal pupil reaction, ISS ≥25, prehospital arterial hypotension and hypothermia.

### Prehospital risk factors for impaired consciousness at 14 days

In the univariate model 5 risk factors were associated with a higher risk of impaired consciousness at 14 days (GCS < 9 in ED, abnormal pupil reaction, ISS ≥25, prehospital hypoxemia and prehospital hypothermia; Table 4); 3 protective factors (age, trauma mechanism other than road traffic accidents, indirect admission) were associated with a lower risk of impaired consciousness at 14 days; not associated were prehospital hypotension, and prehospital time.

**Table 4:** Risk and protective factors of impaired consciousness at 14 days (N = 411, 19 patients without evaluation)

	**Univariate model**		**Multivariate model**	
	**OR (95% CI)**	**P value**	**OR (95% CI)**	**P value**
**Patient-related risks**				
Age	0.99 (0.98-1.00)	0.024	1.00 (0.98; 1.01)	0.837
GCS ≥9 in ED	1		1	
GCS <9 in ED	9.57 (5.34-17.14)	<0.0001	7.01 (3.64; 13.53)	<0.0001
Normal pupil reaction in ED	1		1	
Abnormal pupil reaction in ED	3.07 (1.78-5.29)	<0.0001	1.29 (0.68-2.45)	0.442
ISS <25	1		1	
ISS ≥25	4.54 (2.51-8.20)	<0.0001	2.79 (1.43-5.44)	0.0003
Falls	1		1	
Road traffic accidents	2.05 (1.26; 3.33)	0.004	1.35 (0.72; 2.53)	0.348
Others mechanisms	1.03 (0.52; 2.02)	0.943	0.69 (0.30; 1.63)	0.398
**Treatable prehospital risks**				
Hypotension (syst BP <90)	2.16 (0.67-6.97)	0.196	1.38 (0.29; 6.58)	0.688
Hypoxemia (SpO2 <90%)	5.54 (2.71-11.30)	<0.0001	4.11 (1.72-9.84)	0.001
Hypothermia (<35.0°C)	2.23 (1.33-3.73)	0.002	1.43 (0.77-2.68)	0.259
**Intervention-related risks**				
Prehospital time	1.00 (0.99-1.00)	0.296		
Direct admission	1		1	
Indirect admission	0.37 (0.20-0.68)	0.001	0.47 (0.23-0.97)	0.041

In the multivariate model, 3 risk factors were independently associated with a higher risk of impaired consciousness: GCS <9 in ED, ISS ≥25 and prehospital hypoxemia. One protective factor (indirect admission) was associated with a lower risk of impaired consciousness.

## Discussion

### Main results

One main result of this study was that arterial hypotension and hypothermia are prehospital risk factors for mortality. Another main result was that hypoxemia is a prehospital risk factor for impaired consciousness for survivors at 14 days. In our cohort with a mature emergency medical system, prehospital time was not identified as risk or protective factors for mortality or impaired consciousness of survivors.

### Comparison with earlier studies

Our finding confirms that arterial hypotension is a prehospital risk factor for mortality. In a recent study in the Netherlands including 339 adult patients with a CT-confirmed severe TBI and a GCS ≤8 (mean age: 44 years) similar risks factors including prehospital hypotension were observed; independent predictors were age, ISS, disturbed pupillary reflex and arterial hypotension [17]. A further similarity to our study was that hypoxemia could not be identified as an independent prehospital risk factor for mortality at 14 days; however, prehospital time and hypothermia were not investigated. In an older study Schreiber et al. identified arterial hypotension in ED as an independent risk factor, together with intracranial hypertension, for hospital mortality in 368 adult patients with a HAIS 5 (mean age: 35 years) [29]. In contrast, using the Glasgow Outcome Scale (GOS) at 6 months as outcome, McHugh et al. identified arterial hypotension, hypoxemia and hypothermia prior to or on admission to hospital as independent risk factors for poor outcome based on a meta-analyses of individual data of 7 randomized controlled trials [15].

We observed an association between prehospital hypothermia and mortality. Similarly, a retrospective cohort study based on the Los Angeles Trauma Database observed that in moderate to severe TBI, there was an association between prehospital hypothermia and mortality; however and in contrast to this study, the number of patients with hypothermia was small (n = 44) which decreased the validity of the observed result [30]. Our result is also in line with the meta-analysis of McHugh et al [15].

In our prospective observational study in Switzerland with a well-organized trauma system and a high number of older patients we could not confirm the association between direct admission and mortality. This is in contrast to an analysis of the online database called TBI-trac in New York State including 1123 patients (with GCS ≤8) which observed an association between indirect admission and mortality [31]. The divergence between the studies may be related to the following facts: first, the case-mix was younger (mean age 36 years) with probably more road traffic accidents and, second, the longer times for indirect admission (mean 4.5 h) in the New York State study.

Impaired consciousness 14 days after trauma of survivors is rarely used as outcome and its utilization may extend previous observations. In contrast to the initial period reaching several days after severe TBI, an evaluation with the GCS is probably less biased by interrater variability and changes over time [27]. In our cohort impaired consciousness of survivors was independently associated with unconsciousness in the ED, a higher degree of injury and prehospital hypoxemia. A similar association was observed in a retrospective study including 1547 patients with a HAIS ≥3 and mean age of 41 years where the average PO2 <100 mmHg in the first 24 hours was associated with a worse discharge GCS score [19]. Interestingly, these authors observed the same association with hyperoxia (>200 mmHg in the first 24 hours) suggesting that the therapeutic safety range of oxygen in severe TBI is small.

### Clinical relevance

Isolated severe TBI without bleeding is associated with arterial hypertension related to sympathetic nervous system activation [20,21]; therefore, arterial hypotension is probably most often associated with considerable hypovolemia related to a potentially invisible additional trauma with bleeding contributing to the risk of death. Based on statistical models, it has been proposed that systolic blood pressure should be 110 mmHg to avoid low cerebral perfusion pressure-associated mortality [32]. In another model Butcher et al. proposed an optimal systolic blood pressure of 135 mmHg for best outcome [33]. However, based on a retrospective cohort study using the large American College of Surgeons National Trauma Data Bank, it was hypothesized that prehospital intravenous administration of fluids was independently associated with mortality [34]; the authors discouraged the use of intravenous fluids and proposed a concept of hypotensive resuscitation. Furthermore, there is evidence, based on a randomized controlled trial, that administration of prehospital hypertonic resuscitation in patients with severe TBI and hypotension is not effective [35]. It is possible that a subgroup of patients suffering from severe TBI with hypotension, perhaps older patients, may benefit of fluid and low continuous doses of norepinephrine; however, such treatment protocols have to tested in randomized trials.

Prehospital hypothermia is major challenge for northern countries and countries with high mountains in particular, in winter seasons. Furthermore, hypothermia may be aggravated in intubated and sedated patients on scene. However, only very few concepts of prehospital active warming have been tested [36]. Further research including outcome data are needed for this promising therapeutic approach.

Prehospital hypoxemia after severe TBI may be related to aspiration or to supplementary thorax injuries. Severe chest trauma and aspiration related to blood or gastric content may occur very early after the trauma [37,38], even before OHEMS are on scene and before “protective” intubation or drainage can be performed. Together with absence of evidence [39], the potential benefit of prehospital intubation and ventilation with positive pressure is controversial. It is possible that a subgroup of patients suffering from severe TBI, with hypoxemia at scene of injury or long distances to a trauma center, may benefit from prehospital intubation, ventilation avoiding hyper- and hypocarbia, and limited administration of oxygen.

### Strength of the study

This cohort study with adults suffering from severe TBI is a prospective study with precisely defined outcomes at 14 days with few lost in the follow-up, including different centers in a high income country with a mature trauma system. The data were analyzed with validated methods. Therefore, our findings can be generalized to countries with a similar situation and may be considered as a best-case scenario.

### Limitations of the study

Our results are associations and, therefore, may not be causative. We can’t exclude that some patients with severe TBI were not admitted to a trauma centre and only received treatment in a primary-care hospital against the legal regulation. Patient selection for this cohort was performed by local collaborators from the participating trauma centers, and therefore, some inter-rater variability cannot be excluded; however, data pertaining to inclusion criteria was scrutinized by the data management centre and, in case of a discrepancy, all inclusion criteria were re-checked with the local collaborators. Temperature measurement was not performed in all patients with severe TBI; therefore, we can’t exclude a minor bias of our results including only patients with temperature measurement.

## Conclusions

Prehospital hypotension and hypothermia were associated with short term mortality after serve TBI. Prehospital hypoxemia was associated with impaired consciousness at 14 days.

## Competing interests

The authors declare no conflicts of interest. B.W. received support from the Swiss Accident Company and the Bangerter-Rhyner Foundation, Switzerland, for this project. The founding agencies had no role in the preparation, review or approval of the manuscript.

## Authors’ contributions

MZ, GH, BW were responsible for conception, design, acquisition of data, and successful realization of the multi-centre study. ST and BW did the literature research. C.D, GH BW analyzed the data and interpreted the results in collaboration with ST and MZ. ST wrote the first draft of the manuscript. All authors critically revised the manuscript and approved the final version.

## References

[B1] Mosconi P Taricco M Bergamini M Bosisio Fazzi L Colombo C Patrucco V Corti M Giobbe D Guerreschi M Magnarella MR Sallemi G Family burden after severe brain injury: the Italian experience with families and volunteer associations Patient 2011 4 55 65 10.2165/11535550-000000000-00000 21766894

[B2] Pielmaier L Walder B Rebetez MM Maercker A Post-traumatic stress symptoms in relatives in the first weeks after severe traumatic brain injury Brain Inj 2011 25 259 265 10.3109/02699052.2010.542429 21280978

[B3] Dikman SS Ross BL Machamer JE Temkin NR One year psychosocial outcome in head injury J Int Neuropsy Soc 1995 1 67 77 10.1017/S1355617700000126 9375211

[B4] Patel HC Bouamra O Woodford M King AT Yates DW Lecky FE Trends in head injury outcome from 1989 to 2003 and the effect of neurosurgical care: an observational study Lancet 2005 366 1538 1544 10.1016/S0140-6736(05)67626-X 16257340

[B5] von Elm E Osterwalder JJ Graber C Schoettker P Stocker R Zangger P Vuadens P Egger M Walder B Severe traumatic brain injury in Switzerland - feasibility and first results of a cohort study Swiss Med Wkly 2008 138 327 334 18561037 10.4414/smw.2008.12025

[B6] Kool B Raj N Wainiqolo I Kafoa B McCaig E Ameratunga S Hospitalised and fatal head injuries in Viti Levu, Fiji: findings from an island-wide trauma registry (TRIP 4) Neuroepidemiol 2012 38 179 185 10.1159/000337261 PMC3375116 22472517

[B7] Schirmer-Mikalsen K Vik A Gisvold SE Skandsen T Hynne H Klepstad P Severe head injury: control of physiological variables, organ failure and complications in the intensive care unit Acta Anaesthesiol Scand 2007 51 1194 1201 17711565 10.1111/j.1399-6576.2007.01372.x

[B8] Baguley IJ Nott MT Howle AA Simpson GK Browne S King AC Cotter RE Hodgkinson A Late mortality after severe traumatic brain injury in New South Wales: a multicentre study Med J Aust 2012 196 40 45 22256933 10.5694/mja11.10090

[B9] Rosenfeld JV Maas AI Bragge P Morganti-Kossmann MC Manley GT Gruen RL Early management of severe traumatic brain injury Lancet 2012 380 1088 1098 10.1016/S0140-6736(12)60864-2 22998718

[B10] Sheps Mangulies S Thibault LE Gennarelli TA Physical model simulations of brain injury in the primate J Biomechanics 1990 23 823 836 10.1016/0021-9290(90)90029-3 2384494

[B11] Kalia LV Kalia SK Salter MW NMDA receptors in clinical neurology: excitatory times ahead Lancet Neurol 2008 7 742 755 10.1016/S1474-4422(08)70165-0 18635022 PMC3589564

[B12] Morganti-Kossmann MC Satgunaseelan L Bye N Kossmann T Modulation of immune response by head injury Injury 2007 38 1392 1400 10.1016/j.injury.2007.10.005 18048036

[B13] Cernak I Vink R Zapple DN Cruz MI Ahmed F Chang T Fricke ST Faden AI The pathobiology of moderate diffuse traumatic brain injury as identified using a new experimental model of injury in rats Neurobiol Dis 2004 17 29 43 10.1016/j.nbd.2004.05.011 15350963

[B14] Hellal F Bonnefont-Rousselot D Crocci N Palmier B Plotkine M Marchand-Verrecchia C Pattern of cerebral edema and hemorrhage in a mice model of diffuse brain injury Neurosci Lett 2004 357 21 24 10.1016/j.neulet.2003.12.036 15036604

[B15] McHugh GS Engel DC Butcher I Steyerberg EW Lu J Mushkudiani N Hernandez AV Marmarou A Maas AI Murray GD Prognostic value of secondary insults in traumatic brain injury: results from the IMPACT study J Neurotrauma 2007 24 287 293 10.1089/neu.2006.0031 17375993

[B16] Hukkelhoven CW Steyerberg EW Farace E Habbema JD Marshall LF Maas AI Regional differences in patient characteristics, case management, and outcomes in traumatic brain injury: experience from the tirilazad trials J Neurosurg 2002 97 549 557 10.3171/jns.2002.97.3.0549 12296638

[B17] Franschman G Peerdeman SM Andriessen TM Greuters S Toor AE Vos PE Bakker FC Loer SA Boer C Effect of secondary prehospital risk factors on outcome in severe traumatic brain injury in the context of fast access to trauma care J Trauma 2011 71 826 832 10.1097/TA.0b013e31820cebf0 21427618

[B18] de Ribaupierre S Trauma and impaired consciousness Neurol Clin 2011 29 883 902 10.1016/j.ncl.2011.07.012 22032665

[B19] Brenner M Stein D Hu P Kufera J Wooford M Scalea T Association between early hyperoxia and worse outcomes after traumatic brain injury Arch Surg 2012 147 1042 1046 10.1001/archsurg.2012.1560 22801994

[B20] Hamill RW Woolf PD McDonald JV Lee LA Kelly M Catecholamines predict outcome in traumatic brain injury Ann Neurol 1987 21 438 443 10.1002/ana.410210504 3592639

[B21] Woolf PD Hamill RW Lee LA Cox C McDonald JV The predictive value of catecholamines in assessing outcome in traumatic brain injury J Neurosurg 1987 66 875 882 10.3171/jns.1987.66.6.0875 3572517

[B22] Leon-Carrion J Leon-Dominguez U Pollonini L Wu MH Frye RE Dominguez-Morales MR Zouridakis G Synchronization between the anterior and posterior cortex determines consciousness level in patients with traumatic brain injury (TBI) Brain Res 2012 1476 22 30 22534483 10.1016/j.brainres.2012.03.055

[B23] Kouloulas EJ Papadeas AG Michail X Sakas DE Boviatsis EJ Prognostic value of time-related Glasgow Coma Scale components in severe traumatic brain injury: a prospective evaluation with respect to 1-year survival and functional outcome Int J Rehabil Res 2013 36 260 267 10.1097/MRR.0b013e32835fd99a 23470551

[B24] Walder B Haller G Rebetez MM Delhumeau C Bottequin E Schoettker P Ravussin P Brodmann Maeder M Stover JF Zürcher M Haller A Wäckerlin A Haberthür C Fandino J Haller CS Osterwalder J Severe traumatic brain injury in a high-income country: An epidemiological study J Neurotrauma 2013 30 1934 1942 10.1089/neu.2013.2955 23822874

[B25] Garthe E States JD Mango NK Abbreviated injury scale unification: the case for a unified injury system for global use J Trauma 1999 47 309 323 10.1097/00005373-199908000-00016 10452467

[B26] Patel NY Hoyt DB Nakaji P Marshall L Holbrook T Coimbra R Winchell RJ Mikulaschek AW Traumatic brain injury: patterns of failure of nonoperative management J Trauma 2000 48 367 374 10.1097/00005373-200003000-00001 10744271

[B27] Zuercher M Ummenhofer W Baltussen A Walder B The use of Glasgow Coma Scale in injury assessment: a critical review Brain Inj 2009 23 371 384 10.1080/02699050902926267 19408162

[B28] Copes WS Champion HR Sacco WJ Lawnick MM Keast SL Bain LW The Injury Severity Score revisited J Trauma 1988 28 69 77 10.1097/00005373-198801000-00010 3123707

[B29] Schreiber MA Aoki N Scott BG Beck JR Determinants of mortality in patients with severe blunt head injury Arch Surg 2002 137 285 290 10.1001/archsurg.137.3.285 11888450

[B30] Bukur M Kurtovic S Berry C Tanios M Ley EJ Salim A Pre-hospital hypothermia is not associated with increased survival after traumatic brain injury J Surg Res 2011 175 24 29 21872881 10.1016/j.jss.2011.07.003

[B31] Hartl R Gerber LM Iacono L Ni Q Lyons K Ghajar J Direct transport within an organized state trauma system reduces mortality in patients with severe traumatic brain injury J Trauma 2006 60 1250 1256 10.1097/01.ta.0000203717.57821.8d 16766968

[B32] Berry C Ley EJ Bukur M Malinoski D Margulies DR Mirocha J Salim A Redefining hypotension in traumatic brain injury Injury 2011 43 1833 1837 21939970 10.1016/j.injury.2011.08.014

[B33] Butcher I Maas AI Lu J Marmarou A Murray GD Mushkudiani NA McHugh GS Steyerberg EW Prognostic value of admission blood pressure in traumatic brain injury: results from the IMPACT study J Neurotrauma 2007 24 294 302 10.1089/neu.2006.0032 17375994

[B34] Haut ER Kalish BT Cotton BA Efron DT Haider AH Stevens KA Kieninger AN Cornwell EE 3rd Chang DC Prehospital intravenous fluid administration is associated with higher mortality in trauma patients: a National Trauma Data Bank analysis Ann Surg 2011 253 371 377 10.1097/SLA.0b013e318207c24f 21178760

[B35] Cooper DJ Myles PS McDermott FT Murray LJ Laidlaw J Cooper G Tremayne AB Bernard SS Ponsford J Prehospital hypertonic saline resuscitation of patients with hypotension and severe traumatic brain injury: a randomized controlled trial JAMA 2004 291 1350 1357 10.1001/jama.291.11.1350 15026402

[B36] Lundgren P Henriksson O Naredi P Bjornstig U The effect of active warming in prehospital trauma care during road and air ambulance transportation - a clinical randomized trial Scand J Trauma Resusc Emerg Med 2011 19 59 10.1186/1757-7241-19-59 22017799 PMC3214151

[B37] Lockey DJ Coats T Parr MJ Aspiration in severe trauma: a prospective study Anaesthesia 1999 54 1097 1098 10.1046/j.1365-2044.1999.00754.x 10540100

[B38] Vadeboncoeur TF Davis DP Ochs M Poste JC Hoyt DB Vilke GM The ability of paramedics to predict aspiration in patients undergoing prehospital rapid sequence intubation J Emerg Med 2006 30 131 136 10.1016/j.jemermed.2005.04.019 16567245

[B39] von Elm E Schoettker P Henzi I Osterwalder J Walder B Pre-hospital tracheal intubation in patients with traumatic brain injury: systematic review of current evidence Br J Anaesth 2009 103 371 386 10.1093/bja/aep202 19648153

